# The Burden of Surgical Site Infections With Pathogens Presumably Resistant to Perioperative Prophylaxis in Orthopedic Tumor Surgery: Secondary Analysis of the Prophylactic Antibiotic Regimens in Tumor Surgery (PARITY) Trial

**DOI:** 10.1093/infdis/jiaf513

**Published:** 2025-10-06

**Authors:** Sabine Kuster, Caleb Gottlich, Timothy O’Shea, Michelle Ghert, Dominik Mertz

**Affiliations:** Division of Infectious Diseases, Department of Medicine, McMaster University, Hamilton, Ontario, Canada; Department of Orthopaedic Surgery, Texas Tech University Health Sciences Center, Lubbock, Texas, USA; Division of Infectious Diseases, Department of Medicine, McMaster University, Hamilton, Ontario, Canada; Division of Orthopaedic Surgery, Department of Surgery, McMaster University, Hamilton, Ontario, Canada; Division of Infectious Diseases, Department of Medicine, McMaster University, Hamilton, Ontario, Canada

**Keywords:** resistance, orthopedic, prophylaxis, surgery, infection

## Abstract

**Background:**

Surgical procedures for malignant bone tumors of the lower extremity are associated with a significant risk of surgical site infection (SSI). Little is known about the microbiology and risk factors for resistant SSIs in this population.

**Methods:**

We describe the microbiological and other characteristics and of SSIs, as well as risk factors for antimicrobial resistance against antibiotics used for perioperative prophylaxis in a secondary analysis of the Prophylactic Antibiotic Regimens in Tumor Surgery (PARITY) trial population. The PARITY trial assessed the effects of short-term (24 hour) versus long-term (5-day) postoperative antibiotic prophylaxis on the SSI incidence in orthopedic oncology.

**Results:**

SSIs were identified in 96 of 604 patients (15.9%), with ≥1 pathogen isolated in 73 (76.0%). The most common pathogens were coagulase-negative staphylococci (34.4%), *Staphylococcus aureus* (24.0%), and Enterobacterales (22.9%). The proportions of pathogens with presumed resistance against cephalosporins were similar in the 2 groups (65.9% in the short-term vs 71.9% in the long-term arm; odds ratio [OR], 0.76 [95% confidence interval, .28–2.06]; *P* = .58). Neutropenia (22.9% vs 4.8%; OR, 5.95 [95% confidence interval, .72–49.45]; *P* = .06) and initiation of antibiotics >7 days before SSI diagnosis (50.0% vs 34.8%; OR, 1.88 [.68–5.21; *P* = .22) were numerically but not statistically significantly more common in those with presumed resistance.

**Conclusions:**

SSIs due to pathogens presumably resistant to the systemic or local prophylactic agents used are common in patients undergoing reconstruction for bone tumors. The selection of presumably resistant pathogens is not driven by the duration of antibiotic prophylaxis; however, antibiotic-loaded cement was associated with resistance.

## Teaser

We identified surgical site infections in 16% of patients after bone tumor surgery, primarily due to staphylococci and Enterobacterales. Pathogens resistant to perioperative prophylaxis were frequent but not influenced by the duration of prophylaxis. Antibiotic-loaded cement was associated with resistance.

Patients with malignant tumors of the femur or tibia require a multidisciplinary treatment strategy including surgery and in some cases chemotherapy and/or radiation therapy. Surgical reconstruction is often complex and frequently requires the use of modular endoprostheses to replace the resected bones and joints. Evidence suggests that for elective joint surgery for nononcologic indications, antibiotic prophylaxis lasting <24 hours is as effective as longer durations [1–3]. Therefore, guidelines and consensus statements include this recommendation [4–7]. However, because data on the optimal duration of perioperative antibiotic prophylaxis in orthopedic oncology are scarce, similar prevention strategies are recommended [8, 9]. More recently, the Prophylactic Antibiotic Regimens in Tumor Surgery (PARITY) trial did not show superiority of 5-day versus 1-day postoperative prophylaxis with regard to the incidence of surgical site infections (SSIs) [10].

Morbidity due to SSI in the cohort of patients with bone tumors is substantial and can lead to delayed adjuvant chemotherapy or amputation [11–13]. Compared with patient undergoing elective hip and knee arthroplasty, those undergoing orthopedic surgery of the lower limbs due to cancer have an increased risk of SSIs [8, 14–16]. SSI rates are reported to be in the range of 1%–3% for elective hip and knee arthroplasty [17, 18], while rates in orthopedic oncology are reported to be on 10% average and as high as 36% [8, 10, 13–16]. The higher SSI rate in tumor orthopedic surgery is likely related to the patient population itself and the administration of chemotherapy and/or radiation therapy predisposing for difficulties with wound healing. Some retrospective studies and a review article showed that pathogens isolated in SSIs in this population often harbor cephalosporin-resistant pathogens, such as coagulase-negative staphylococci, gram-negative bacilli, and enterococci [8, 13, 15, 19]. There is also some evidence suggesting that prolonged duration of postoperative antibiotic prophylaxis increases of risk for infections due to resistant bacteria [20].

Using the data from the PARITY trial, the primary goal of the present study was to characterize the microbiological characteristics in SSIs in this population as well as the presumed resistance to the perioperative prophylactic agents used. We also aimed to identify risk factors associated with the isolation of presumably resistant pathogens. Finally, we assessed the association between topical antibiotics, such as antibiotic cement and locally applied powder/sponge, and the selection of presumably resistant pathogens.

## METHODS

### Data Set

Designed as a multicenter blinded randomized controlled trial with 2 parallel study arms, the PARITY trial assessed the effect of short-term (24-hour) versus long-term (5-day) postoperative intravenous antibiotic prophylaxis with cefazolin or cefuroxime (clinical site specific) on the incidence of SSI in patients aged ≥12 years undergoing surgical excision and endoprosthetic reconstruction due to a bone tumor of the lower limb [10, 21]. Patients at 48 clinical sites in North America, South America, Europe, Australia, Africa, and Asia were randomized in a 1:1 ratio to the study arms and followed up for 1 year postoperatively. Further details of the study design have been published elsewhere [21]. The PARITY was approved by the Hamilton Integrated Research Ethics Board as well as the relevant local ethics committee at each participating site. The current study was performed using the PARITY dataset to further analyze the microbiological characteristics of SSI in this patient population.

### Microbiological Sampling

Microbiological sampling was considered only if it was performed in an aseptic manner (eg, tissue cultures, joint fluid, deep swab samples, and sonication of prosthesis), in accordance with the Centers for Disease Control and Prevention (CD)C/NSHN definition of SSIs [22]. Blood cultures taken at the time of SSI diagnosis were documented and considered alongside the other culture results. Microbiological cultures and resistance testing were performed according to local standards at each study site.

### Outcomes

The primary end point of this secondary analysis was to characterize the proportion and microbiological spectrum of SSIs caused by pathogens presumably resistant to cefazolin or cefuroxime prophylaxis. The secondary end point was to identify potential risk factors for SSIs caused by pathogens presumably resistant to standard prophylaxis. In addition, the rate of pathogens presumably resistant to topic antibiotics was assessed.

SSIs were classified based on the 1992 Centers for Disease Control and Prevention (CDC)/National Healthcare Safety Network (NHSN) definitions of nosocomial SSI [22]. Superficial incisional SSIs were included if the onset of symptoms was within 30 days after surgery, whereas deep and/or organ space SSIs were considered if the onset was within a full year after surgery, with the surveillance period starting on the date of the initial surgery. In patients with recurrent infections, only the first SSI episode diagnosed was included in the current analysis.

A pathogen was deemed relevant if the antibiotic regimen administered to treat the SSI episode provided adequate coverage. Presumed resistance to the prophylactic agent was defined considering reported susceptibility data where available. If these data were unavailable, we considered known intrinsic resistance mechanisms for a given pathogen to determine resistance and/or inferred presumed resistance from the antibiotics chosen to treat the SSI episode. If no susceptibility data were available for coagulase-negative staphylococci, the isolate was deemed to be methicillin resistant. For polymicrobial infections, the SSI was classified as resistant if ≥1 relevant pathogen was presumably resistant to cefazolin or cefuroxime. The same approach was used for resistance to antibiotics used in antibiotic cement and antibiotic powder/sponges.

### Patient Cohorts and Risk Factors for Presumed Resistance

We conducted 2 sensitivity analyses. First, we reassessed all SSI using the most up-to-date and more stringent 2024 CDC/NHSN surveillance definition [23]. Second, we excluded patients from 1 site in India; this site enrolled 85 of 604 patients (14.1%) and routinely prescribed preemptive antibiotic therapy immediately after completion of the 24-hour or 5-day PARITY prophylaxis.

We defined antibiotic exposure as a risk factor for the selection of resistant pathogens, regardless of treatment duration, if initiated >7 days before the SSI diagnosis. This was under the assumption that antibiotic treatment initiated within 7 days of the eventual SSI diagnosis was unlikely to contribute to the risk of resistance in an SSI already in development.

### Statistical Analysis

Data were first analyzed descriptively. For comparisons between groups of categorical data, we used χ^2^ or Fisher exact tests as appropriate, and we reported odds ratios (ORs) with corresponding 2-sided 95% confidence intervals (CIs). Independent sample *t* tests were used to compare continuous variables between groups. For multivariate analysis, we used binary logistic regression to identify potential independent risk factors for presumably resistant SSI using a conditional backward modeling approach. Factors chosen for our model have either been previously identified as risk factors for SSI in tumor orthopedic surgery (eg, absolute neutrophil count <1500/μL at initial surgery, administration of chemotherapy and/or radiotherapy before surgery, or revision surgery) or could have been potential confounders (eg, previous antibiotic exposure or use of local antibiotics). All statistical tests were performed using 2-sided tests at the .05 level of significance. All analyses were performed using SPSS/PASW software, version 29 (IBM Corp. (2023)).

## RESULTS

Of 604 patients in the PARITY trial, 96 (15.9%) were diagnosed with an SSI. Diagnosis of deep/organ space SSI was documented in 75.0% of episodes (n = 72), and superficial SSIs were reported in 25.0% (n = 24). The majority of the patients were male (n = 57 [59.4%]) and white (n = 59 [61.5%]), and the average age was 39.7 years (Table 1). Primary bone sarcoma was the most common indication for surgery. The majority of patients had tumors involving the femur.

**Table 1.** Baseline Characteristics of Patients With Surgical Site Infections Diagnosed

Characteristic	Patients With SSIs, No. (%)^a^		
	All Patients (n = 96)	Short-Term Antibiotic Prophylaxis (n = 52)	Long-Term Antibiotic Prophylaxis (n = 44)
Age, mean (SD), y	39.7 (23.8)	35.9 (23.9)	40.4 (23.9)
Female sex	39 (40.6)	20 (38.5)	19 (43.2)
Race/ethnicity			
White	59 (61.5)	30 (57.7)	29 (65.9)
Asian	15 (15.6)	11 (21.2)	4 (9.1)
Black	7 (7.3)	2 (3.8)	5 (11.4)
Hispanic	9 (9.4)	5 (9.6)	4 (9.1)
Native	6 (6.3)	4 (7.7)	2 (4.5)
Type of tumor			
Bone tumor	81 (84.4)	43 (82.7)	38 (86.4)
Soft-tissue sarcoma	12 (12.5)	7 (13.5)	5 (11.4)
Oligometastatic bone disease	3 (3.1)	2 (3.8)	1 (2.3)
Systemic metastases	17 (17.7)	11 (21.2)	6 (13.6)
Location of tumor			
Tibia	22 (22.9)	11 (21.2)	11 (25.0)
Femur	74 (77.1)	41 (78.8)	33 (75.0)
Preoperative cancer treatment			
No	51 (53.1)	24 (46.2)	27 (61.4)
Chemotherapy	45 (46.9)	28 (53.8)	17 (38.6)
Radiation therapy	6 (6.3)	4 (7.7)	2 (4.5)
Other	2 (2.1)	2 (3.6)	0 (0)
Neutropenia (ANC <1500/μL) at time of surgery (data missing in 4 patients)	14 (15.2)	7 (14.0)	7 (16.7)
Type of skin disinfection			
Iodine	21 (21.9)	12 (23.1)	9 (20.5)
Alcohol	25 (26.0)	13 (25.0)	12 (27.3)
Chlorhexidine	85 (88.5)	46 (88.5)	39 (88.6)
Duration of surgery, mean (SD), min	371.7 (167.9)	362.5 (163.1)	382.4 (173.0)
Use of antibiotic cement	55 (57.3)	30 (57.7)	25 (56.8)
Use of antibiotic sponge/powder	16 (16.7)	11 (21.2)	5 (11.4)
Bacterial pathogens detected			
Presumed susceptible to prophylaxis	23 (24.0)	14 (26.9)	9 (20.5)
Presumed resistant to prophylaxis	50 (52.1)	27 (51.9)	23 (52.3)
No pathogens	23 (24.0)	11 (21.2)	12 (27.3)

Abbreviations: ANC, absolute neutrophil count; SSI, surgical site infection.

^a^Data represent no. (%) of patients unless otherwise specified.

The most frequent surgical intervention performed at the time of SSI diagnosis was implant irrigation and debridement (n = 51 [79.8%]), which included exchange of mobile parts in 35.3% (n = 18), followed by implant removal in 13.9% (n = 10), 1- or 2-stage implant exchanges in 9.7% (n = 7), and amputation in 2.8% (n = 2). Diagnosis of deep/organ space SSI was based on clinical findings only in 2.8% (n = 2). On average, 3 cultures were sent for microbiological analysis (range, 0–15 cultures; SD, 2.6).

A total of 73 of 96 patients (76.0%) had a culture-positive SSI. Among these patients, deep/organ space SSIs were diagnosed in 87.7% (n = 64) and superficial SSIs in 12.3% (n = 9). At least 1 pathogen was deemed relevant in 70 cases (96%), consisting of 6 superficial and 64 deep/organ space SSI episodes. Polymicrobial infections were present in 30 of these patients (42.9%), including 29 with deep/organ space SSIs. The 4 most frequently detected pathogens were coagulase-negative staphylococci in 34.4%, *Staphylococcus aureus* in 24.0% (methicillin-susceptible and methicillin-resistant isolates), Enterobacterales in 22.9%, and nonfermenters in 18.8% (Table 2).

**Table 2.** Microbiological Spectrum of 96 Surgical Site Infection Episodes

Pathogen	All SSIs, No. (%) (n = 96)^a^	Isolates Deemed Relevant, No. (%)	Deep/Organ Space SSIs, No. (%)	
			Total (n = 72)^b^	Isolates Deemed Relevant
Gram-positive bacteria	95 (98.9)	76 (80.0)	92 (127.8)	73 (79.3)
MSSA	17 (17.7)	17 (100.0)	16 (17.4%)	16 (100.0)
MRSA	6 (6.3)	6 (100.0)	5 (6.9)	5 (100.0)
* Staphylococcus lugdunensis*	3 (3.1)	3 (100.0)	3 (4.2)	3 (100.0)
* Coagulase-negative* staphylococci^c^	33 (34.4)	25 (75.8)	32 (44.4)	24 (75.0)
* Streptococcus* spp.	8 (8.3)	8 (100.0)	8 (11.1)	8 (100.0)
* Enterococcus* spp.^d^	14 (14.6)	11 (78.6)	14 (19.4)	11 (78.6)
Aerobic	8 (8.3)	4 (50.0)	8 (11.1)	4 (50.0)
Anaerobic	6 (6.3)	2 (33.3)	6 (8.3)	2 (33.3)
Gram-negative bacteria	45 (46.9)	41 (91.1)	39 (51.2)	37 (94.9)
Enterobacterales^e^	22 (22.9)	22 (100.0)	19 (26.4)	19 (100.0)
* Campylobacter showae*	1 (1.0)	1 (100.0)	1 (1.4)	1 (100.0)
* Bacteroides* spp.	4 (4.2)	4 (100.0)	4 (5.5)	4 (100.0)
Nonfermenter^f^	18 (18.8)	14 (77.8)	15 (20.8)	13 (86.7)
*Candida* spp.	5 (5.2)	1 (20.0)	5 (6.9)	1 (20.0)
No pathogen	23 (24.0)	NA	8 (11.1)	NA

Abbreviations: MRSA, methicillin-resistant *Staphylococcus aureus*; MSSA, methicillin-resistant *S. aureus*; NA, not applicable; SSI, surgical site infection.

^a^The total is >100%, as 30 patients (31.3%) had polymicrobial infections.

^b^The total is >100%, as 29 patients (40.3%) had polymicrobial infections.

^c^The most commonly isolated species was *Staphylococcus epidermidis* (n = 17 [51.5%]), followed by not-further-specified coagulase-negative staphylococci (n = 11 [36.4%]) and *Staphylococcus haemolyticus* (12.1%).

^d^Among enterococci, *Enterococcus faecalis* (n = 8, 57.1%) was the most common species.

^e^Infections caused by Enterobacterales were driven by AmpC-producing bacteria (n = 10 [45.5%]; eg, *Enterobacter cloacae*) followed by *Escherichia coli* (n = 7 [31.8%]).

^f^ *Pseudomonas aeruginosa* accounted for 61.1% of infections (n = 11) with nonfermenters followed by *Acinetobacter* spp. (n = 4 [22.2%]).

In culture-positive SSI, data on susceptibility testing were available in 38.4% (n = 28). Presumably resistant pathogens were detected in 68.5% of SSI cases (50 of 73 SSIs with ≥1 pathogen isolated). There was no statistically significant difference in the occurrence of resistant pathogens when comparing the 2 groups, with 65.9% (27 of 41) in the short-term and 71.9% (23 of 32) in the long-term prophylaxis group, respectively (OR, 0.76 [95% CI, .28–2.06]; *P* = .58). SSIs caused by presumably cephalosporin-resistant pathogens occurred a mean (SD) of 73.6 days (97.6) days after index surgery, compared with 105.2 (107.2) days for infections with susceptible pathogens (mean difference, −31.58 days [95% CI, −82.14 to 18.99]; *P* = .22). Antibiotic treatment for any cause was initiated earlier in patients with resistant SSIs (at a mean [SD] of 32.7 [45.7] days) than in those with cephalosporin-susceptible pathogens (at 65.3 [90.5] days), although this difference was not significant (mean difference, 19.94 days [95% CI, −73.49 to 8.28]; *P* = .11).

The odds of infection with presumably resistant pathogens numerically higher in patients with absolute neutrophil counts <1500/μL at initial surgery (adjusted OR, 8.09, [95% CI, .93­–70.12]; *P* = .058) and in those exposed to antibiotics >7 days before SSI diagnosis (2.50 [.82–­7.63]; *P* = .11), although these differences are not statistically significant. Patients who underwent preoperative chemotherapy had significantly lower odds of infection with presumably resistant pathogens (adjusted OR, 0.33 [95% CI, .12–.92]; *P* = .03). Table 3 shows the results of the univariate and multivariate analyses to identify possible risk factors for presumably resistant SSI. Integrating the use of antibiotic powder/sponge, reoperation before SSI diagnosis, diabetes mellitus, and radiation therapy before surgery resulted in an unstable multivariate model, presumably due to low event numbers, and were excluded.

**Table 3.** Univariate and Multivariate Risk Factor Analyses for the Development of Surgical Site Infection Pathogens Presumably Resistant Against Perioperatively Used Cephalosporins

Risk Factor^a^	Patients, No. (%)		Univariate Analysis		Multivariate Analysis	
	SSI With Presumably Resistant Pathogens (n = 50)	SSI With Presumably Susceptible Pathogens (n = 23)	OR (95% CI)	*P* Value	OR (95% CI)	*P* Value
Duration of prophylaxis (short vs long)	27 (54.0)	14 (60.9)	0.76 (.28–2.06)	.58	…	…
Antibiotic cement	28 (56.0)	12 (52.2)	1.17 (.43–3.14)	.76	…	…
Antibiotic sponge/powder	5 (10.0)	5 (21.7)	0.40 (.10–1.55)	.18	…	…
Postoperative antibiotics >7 d before SSI	25 (50.0)	8 (34.8)	1.88 (.68–5.21)	.22	2.50 (.82–7.63)	.11
Reoperation before SSI	8 (16.0)	2 (8.7)	2.00 (.39–10.27)	.40	…	…
Diabetes mellitus	2 (4.0)	1 (4.3)	0.92 (.08–10.65)	>.99	…	…
ANC <1500/μL at initial surgery	11 (22.9)	1 (4.8)	5.95 (.72–49.45)	.06	8.09 (.93–70.12)	.06
Chemotherapy before surgery	17 (34.0)	14 (60.9)	0.331 (.12–.92)	.03^b^	…	…
Radiation therapy before surgery	3 (6.0)	1 (4.3)	1.404 (.14–14.28)	>.99	…	…

Abbreviations: ANC, absolute neutrophil count; CI, confidence interval; OR, odds ratio; SSI, surgical site infection.

^a^Except where otherwise noted (for duration of prophylaxis), the comparison was yes versus no.

^b^Significant at *P* < .05.

Sensitivity analyses performed for both modified SSI cohorts confirmed the trends, without reaching statistical significance (Supplementary Tables 1 and 2). The first sensitivity analysis included 68 of 96 patients (70.8%) who met the more stringent 2024 SSI definition. The primary reason for not meeting the updated CDC definitions for superficial SSI (eg, SSI diagnosis beyond day 30 after surgery, treatment for cellulitis/stitch abscess or missing clinical criteria). After exclusion of patients recruited at 1 center in India in the second sensitivity analysis, 85 of 96 patients with an SSI (88.5%) remained in the second modified cohort.

Among the 96 patients with SSIs diagnosed, antibiotic cement was used in 57.3% (n = 55) and antibiotic powder/sponges in 16.7% (n = 16). The rate of cephalosporin-resistant SSI was 70% (n = 28) with local antibiotics and 66.7% (n = 22) without. In patients with aminoglycoside-containing cement, presumably aminoglycoside-resistant pathogens were isolated in 66.7% (32 of 48), whereas no presumably resistant pathogens were detected with use of a macrolide-polymyxin combination (n = 7) (OR, 2.0 [95% CI, 1.0–3.99]; *P* < .001). Table 4 summarizes the topical antibiotics used and the proportion of presumably resistant pathogens, depending on the antibiotic class used.

**Table 4.** Rates of Presumed Resistance to the Topical Antibiotics Used

Topical Antibiotic and Resistance Rates	Patients Evaluated, No. (%)
Antibiotic cement used	55/96 (57.3)
Aminoglycoside cement	48 (87.3)
Pathogen presumably resistant	32 (66.7)
Pathogen presumably susceptible	4 (8.3)
No pathogen identified	12 (25.0)
Macrolide/polymyxin cement	7 (12.7)
Pathogen presumably resistant	0
Pathogen presumably susceptible	4 (57.1)
No pathogen identified	3 (42.9)
Antibiotic sponge/powder used	16/96 (16.7)
Aminoglycoside sponge/powder	2 (12.5)
Pathogen presumably resistant	1 (50.0)
Pathogen presumably susceptible	1 (50.0)
Glycopeptide sponge/powder	14 (87.5)
Pathogen presumably resistant	6 (42.9)
Pathogen presumably susceptible	2 (14.3)
No pathogen identified	6 (42.9)
Antibiotic cement and sponge used	13/96 (13.5)

## DISCUSSION

Gram-positive bacteria, such as coagulase-negative staphylococci and *S. aureus,* were deemed relevant pathogens in 68.8% of SSI cases, whereas Enterobacterales or nonfermenters were presumably relevant in 51.4%. Polymicrobial SSIs were common (42.9%). The proportions of presumed resistance against cephalosporins that are typically used for perioperative prophylaxis were high, 65.9% in the short-term and 71.9% in the long-term arm. There was a numerically increased risk for SSI with presumably cephalosporin-resistant pathogens in patients with neutropenia (absolute neutrophil count <1500/μL) at the time of surgery and in those with antibiotic courses initiated >7 days before SSI diagnosis, although the differences were not statistically significant. Short-term antibiotic prophylaxis was numerically less likely to be associated with presumably resistant SSI. However, all findings were not significant, most likely due to the number of events resulting in insufficient power to detect clinically relevant differences. Aminoglycosides were the most common antibiotics used to load cement, and presumably aminoglycoside-resistant pathogens were frequently identified in this subset of patients. This is in keeping with a previously published study showing an association between exposure to aminoglycoside-loaded cement and an increased risk of prosthetic joint infections due to aminoglycoside-resistant pathogens [24].

There are differences between the bacterial pathogens causing SSI in the PARITY trial and the microbiological spectrum typically detected in SSIs after elective hip and knee arthroplasty. In both populations, gram-positive pathogens such as coagulase-negative staphylococci and *Staphylococcus aureus* are responsible for the majority of SSIs. Infections due to gram-negative pathogens, however, are reported to account for approximately 10% of SSI in elective hip and knee replacement surgery only [13, 15, 25–28], and polymicrobial infection rates range between 10% and 24% [29, 30]. In contrast, we found these rates to be 58.6% and 42.9%, respectively, in our study. Similarly, previously published data suggested that gram-negative pathogens are relevant in 20% to 70% of SSI cases in this population, and polymicrobial infections accounted for 25%–70% of SSI episodes in tumor orthopedic surgery [8, 13, 15, 31]. However, whether broadening of antibiotic prophylaxis could decrease the SSI rates remains to be determined. A prospective, randomized controlled phase 3 trial comparing the effectiveness of cefuroxime or vancomycin-gentamicin as perioperative prophylaxis in tumor and infected orthopedic surgery patients pretreated with antibiotics is underway to address this question [32].

Tumor localization, longer duration of surgery, presence of postoperative wound complications, need for revision surgery, older age, male sex, chemotherapy, and radiation therapy are associated with an increased SSI risk in tumor orthopedic surgery [19, 33–38]. So far, risk factors for SSI with presumably resistant pathogens have not been well characterized. We found a numerically increased risk for SSI with presumably resistant pathogens in patients with absolute neutrophil counts <1500/μL at the time of surgery and in those in whom antibiotic treatment was initiated >7 days before the diagnosis of an SSI. Neutropenia could be a surrogate marker for previous exposure to antibiotics, thus increasing the risk for the selection of resistant pathogens. This explanation is plausible, as it has been shown that an additional day of antibiotics increases the absolute risk for carriage of resistant gram-negative pathogens by 7%, in a meta-analysis from mixed study populations comparing short- versus long-term antibiotic treatment for urinary tract infections, otitis media, and neonatal late-onset sepsis [39].

The evidence on the effectiveness of antibiotic-loaded cement for preventing SSI is inconsistent. A randomized controlled trial in 36 patients with orthopedic tumors found that gentamicin-loaded cement was effective in preventing an SSI [40]. However, other studies found no decrease in infection rates when antibiotic-loaded cement was used [41, 42]. Antibiotic-loaded cement was used in 57.3% of patients with an SSI diagnosed in the PARITY cohort. Interestingly, pathogens presumably resistant to the antibiotic used to load the cement were present in the majority of culture-positive SSIs. This finding is in line with the literature, showing increasing minimal inhibitory concentrations or higher resistance rates to the antibiotic used in antibiotic-loaded cement [43]. While the decision to (not) use topical antibiotics was not specifically studied here, the practice was primarily driven by local standards of care.

Different factors, such as type of cement, cement porosity, antibiotic concentration, and mixing technique, may affect antibiotic elution from antibiotic-loaded cement [44–46]. Either commercially available or customized antibiotic cement is used in the context of joint replacement surgery. Antibiotic concentrations between 5% and 20% seem to be standard for most cements used [44, 46, 47]. Locally applied antibiotics tend to achieve high levels within the compartment where they are used, effectively inhibiting growth of susceptible pathogens while simultaneously selecting for resistant pathogens. Regarding antibiotic elution, in vivo studies suggest that sufficient release is provided for up to 6 months [44–47]. However, it has been shown that the presence of local bone necrosis in the periprosthetic tissue combined with low oxygen concentration reduces the concentration and effect of local antibiotics, especially aminoglycosides [43, 46].

The main strength of this secondary analysis is that we used data from the PARITY trial, which used standardized eligibility criteria, prophylaxis protocol, and follow-up. Our secondary analysis has several limitations, however. First, the PARITY trial was not designed to assess the effect of antibiotic prophylaxis on the selection of resistant pathogens causing SSIs, so detailed antibiotic susceptibility data was often not collected. About 1 in 4 SSI episodes was culture negative, further reducing the total number of patients available for analysis. In 20.8% of deep/organ space SSIs only 1 culture was documented, which met the CDC/NHSN definition used in the study but may not have necessarily triggered treatment in the clinical context especially for low virulent pathogens. While ours is one of the largest cohorts of patients with orthopedic tumors and SSI, the number of events was still limited, preventing us from drawing definite conclusions on potential risk factors and associations.

In conclusion, this secondary analysis of the PARITY trial found that presumed resistance to perioperative prophylactic agents and/or topical antibiotics is common in SSIs in patients with soft-tissue and bone tumors undergoing oncologic orthopedic surgery. The selection of presumably resistant pathogens is not driven by the duration of perioperative antibiotic prophylaxis; however, use of antibiotic-loaded cement was associated with selection of resistant pathogens. Several risk factors potentially contributing to the selection of presumably resistant pathogens were identified; however, the relatively low number of events limited our ability to draw definite conclusions. Further research is needed to determine the effect of topical antibiotics with regard to the selection of resistant pathogens causing SSI.

## Supplementary Material

jiaf513_Supplementary_Data

## Supplementary Data

Supplementary materials are available at *The Journal of Infectious Diseases* online (http://jid.oxfordjournals.org/). Supplementary materials consist of data provided by the author that are published to benefit the reader. The posted materials are not copyedited. The contents of all supplementary data are the sole responsibility of the authors. Questions or messages regarding errors should be addressed to the author.
