# Supplementary material for: The Burden of Surgical Site Infections With Pathogens Presumably Resistant to Perioperative Prophylaxis in Orthopedic Tumor Surgery: Secondary Analysis of the Prophylactic Antibiotic Regimens in Tumor Surgery (PARITY) Trial
Source: J Infect Dis. 2025 Oct 6;233(1):e174–82. doi: 10.1093/infdis/jiaf513 (PMC12811854; doi:10.1093/infdis/jiaf513)
Supplement: jiaf513_Supplementary_Data [file jiaf513_supplementary_data.docx]

**Table S1:** Univariate and multivariate risk factor analyses for the development of SSI with pathogens presumably resistant against perioperatively used cephalosporins (stringent 2024 CDC/NHSN outbreak definition)

|  | SSI with presumably resistant pathogen (n=41) | SSI with presumably susceptible pathogen (n=21) | Univariate analysis | | Multivariate analysis | |
| --- | --- | --- | --- | --- | --- | --- |
|  | *No. of patients (n, %)* | | Odds ratio (95% CI) | p-value | Odds ratio (95% CI) | p-value |
| Duration of prophylaxis (short versus long) | 21/41 (51.2) | 13/21 (61.9) | 0.65 (0.22, 1.89) | 0.424 |  |  |
| Antibiotic cement | 22/41 (53.7) | 10/21 (47.6) | 1.27 (0.44, 3.65) | 0.652 |  |  |
| Antibiotic sponge/powder (yes versus no) | 5/41 (12.2) | 5/21 (23.8) | 0.44 (0.11, 1.75) | 0.285 |  |  |
| Post operative antibiotics >7 days prior SSI | 21/41 (51.2) | 7/21 (33.3) | 2.10 (0.70, 6.28) | 0.180 | 3.16 (0.86, 11.62) | 0.083 |
| Re operation prior to SSI | 8/41 (19.5) | 1/21 (4.8) | 4.85 (0.56, 41.7) | 0.150 |  |  |
| Diabetes mellitus | 2/41 (4.9) | 1/21 (4.8) | 1.03 (0.09, 12.01) | 1.0 |  |  |
| Absolute neutrophile count <1500/mm3 | 9/39 (23.1) | 1/19 (5.3) | 5.40 (0.63, 46.22) | 0.142 |  |  |
| Chemotherapy before surgery | 14/41 (34.1) | 13/23 (61.9) | 0.32 (0.11, 0.95) | 0.037 | 0.23 (0.07, 0.82) | 0.023 |
| Radiation prior to surgery | 3/41 (7.3) | 1/23 (4.8) | 1.58 (0.15, 16.18) | 1.00 |  |  |

**Table S2:** Univariate and multivariate risk factor analyses for the development of SSI with pathogens presumably resistant against perioperatively used cephalosporins (exclusion of patients from site in India)

|  | SSI with presumably resistant pathogen (n=45) | SSI with presumably susceptible pathogen (n=21) | Univariate analysis | | Multivariate analysis | |
| --- | --- | --- | --- | --- | --- | --- |
|  | *No. of patients (n, %)* | | Odds ratio (95% CI) | p-value | Odds ratio (95% CI) | p-value |
| Duration of prophylaxis (short versus long) | 23/45 (51.1) | 12/21 (57.1) | 0.78 (028, 2.23) | 0.647 |  |  |
| Antibiotic cement | 22/45 (48.9) | 10/21 (47.6) | 1.15 (0.41, 3.24) | 0.792 |  |  |
| Antibiotic sponge/powder (yes versus no) | 0/45 (0) | 3/21 (14.3) | 1.17 (0.98, 1.39) | 0.029 |  |  |
| Post operative antibiotics >7 days prior SSI | 21/45 (46.7) | 6/21 (28.6) | 2.19 (0.72, 6.66) | 0.164 | 3.55 (0.97, 13.03) | 0.056 |
| Re operation prior to SSI | 7/45 (15.6) | 2 (9.5) | 1.75 (0.33, 9.25) | 0.707 |  |  |
| Diabetes mellitus | 2/45 (4.4) | 1/21 (4.8) | 0.93 (0.08, 10.87) | 1.0 |  |  |
| Absolute neutrophile count <1500/mm3 | 11/43 (25.6) | 1/19 (5.3) | 6.19 (0.74, 51.91) | 0.085 | 5.12 (0.54,48.73) | 0.155 |
| Chemotherapy before surgery | 14/45 (31.1) | 13/21 (61.9) | 0.28 (0.09, 0.82) | 0.018 | 0.32 (0.09, 1.13) | 0.078 |
| Radiation prior to surgery | 3/45 (6.7) | 1/21 (4.8) | 1.43 (0.14, 14.61) | 1.00 |  |  |
